# Cultural adaptation of a self-review tool for health promoting universities in Bulgaria

**DOI:** 10.3389/fpubh.2024.1399793

**Published:** 2024-06-19

**Authors:** Petya Boncheva, Klara Dokova

**Affiliations:** Department of Social Medicine and Health Care Organization, Faculty of Public Health, Medical University “Prof. Dr. Paraskev Stoyanov”, Varna, Bulgaria

**Keywords:** health promoting universities and colleges, self-review tool, health promotion, healthy settings, cross-cultural adaptation

## Abstract

**Introduction:**

The Health Promoting University initiative is unknown in Bulgaria, and the health promotion potential of Bulgarian universities has not been studied. In order to examine it, a suitable instrument is needed. The UK Healthy Universities Network provides an accessible Self-Review-Tool (SRT). Aim: To present the process of cultural adaptation of the SRT in Bulgarian language.

**Methods:**

The standardized WHO methodology for cultural adaptation of instruments was followed in four stages: (1) Two language translations of the instrument into Bulgarian were made; (2) An expert Delphi discussion reached a consensus on specific health promoting (HP) terms, followed by a backward translation; (3) Pilot testing of the tool among university community representatives was conducted through a survey among a small sample, with independent responses to the SRT questionnaire followed by cognitive interviews; (4) Final revision of the instrument.

**Results:**

Ten public health experts reached a consensus on the name of the initiative and various HP terms. Ten other respondents pre-tested the tool. Difficulties in responding the SRT concerned the meaning of some HP terms, complex words, the system of answers, limited applicability of some statements. Changes were made to 61 of the total 68 elements in the SRT.

**Conclusion:**

All stages of the cultural adaptation were important for the final result. The adapted Bulgarian version of the SRT would be useful to Bulgarian universities that want to make a clear commitment to improving the health of their university community and the wider society.

## 1 Introduction

Health Promoting Universities (HPUs) have proven to be effective settings for improving the health of the university communities—students, faculty, staff and the wider public over the last three decades (1–3). The HPU initiative has evolved into a global movement with regional, national and international networks established. These networks foster collaboration between HPUs across countries, regions and globally, providing valuable resources: strategic documents, action frameworks, practical application experience, good examples, case studies and assessment tools (2, 4–6).

To achieve designation as an HPU, the application of a “whole university approach” to health is a key prerequisite (1, 7, 8). This involves integrating health in all aspects of the institution’s life, making it a core value of the university culture, and implementing processes and policies aimed at improving the health of the entire university community through activities at all possible levels (8).

The scientific literature reveals various methods by which HPUs evaluate their efforts and the effectiveness of their activities while striving to achieve “whole university” involvement. Typically HPUs use self-designed questionnaires, focusing on specific health issues such as students’ risky health behaviors or health awareness. Additionally, interviews assessing health promotion (HP) activities are conducted with staff or representatives of the HPU initiative coordinators (3, 9, 10). These approaches measure the effectiveness of isolated actions among specific university groups under the HP label.

Given that the “whole university approach” is fundamental to the initiative, comprehensive assessment is necessary (11). In this process, the university should be viewed as a unified entity, and the assessment should encompass the health and behavior of people, the conditions of the university environment, and the built-in organizational structures. The involvement of all people of the institution in the planning and implementation of initiatives improving health and wellbeing is one of the main principles of HPU, and evaluating the participation of the entire university community aims to hear the voice of students, lecturers and staff (11, 12). A number of HPUs have conducted such complex assessments, while those that have not cite the absence of a suitable instrument as a barrier (10).

The UK (13) and the Chilean (14) networks provide questionnaires for comprehensive assessment of the HPUs. The German (15) and North American (16) networks have their own good practice standards. One of the tools that is freely available and therefore used by universities around the world is the UK Healthy Universities Network Self-Review Tool (13). It has been developed for use in all stages of institutional self-assessment.

The HPUs initiative is new to Bulgaria, and the potential of the Bulgarian higher education institutions as health promoting settings has not been studied. However international experience has proved that successful and sustainable implementation of the HPU initiatives depends on their adaptation to local needs, traditions and cultures of the respective countries (3, 9, 17). Earlier studies of HP initiatives and policies’ transfer in Bulgaria reveal that international HP programs and documents, created in western environments, in English language may be more effective if culturally adapted to the specific Bulgarian context and language, which applies to all HP documents and instruments (18).

The aim of the present work is to present the process of cultural adaptation of the HPUs’ Self-Review Tool into Bulgarian.

## 2 Materials and methods

### 2.1 The HPU self-review-tool

The original Self-Review Tool (SRT) is a resource of the UK Healthy Universities Network, developed and applied for assessing the whole system approach to health in the university environment (13). The tool is grounded in strategic HPU documents and criteria (11). It can be applied at all stages of HPU initiative implementation, for both initial and continuous self-assessment. The SRT generates a tabular color image of a “traffic light” type, indicating the degree of fulfillment of the HPU criteria. Green signifies over 70% fulfillment of HPU criteria, yellow indicates partial fulfillment (between 45 and 69%) and red indicates insufficient fulfillment (below 45%) signaling a need for future actions. It is recommended that the instrument be completed by a university team representing the main target groups—students, academic, administrative and management staff—to ensure the voice of the entire university community is “heard” (11). The original instrument is in English, consisting of 68 statements, grouped into five sections reflecting the key HP areas: Leadership and governance; Service provision; Facilities and environment; Communication, information and marketing; Academic, personal, social and professional development. Structured standard responses are provided after each statement, and respondents may also provide free text after each statement and section (13).

### 2.2 The cultural adaptation process

**Figure 1:**
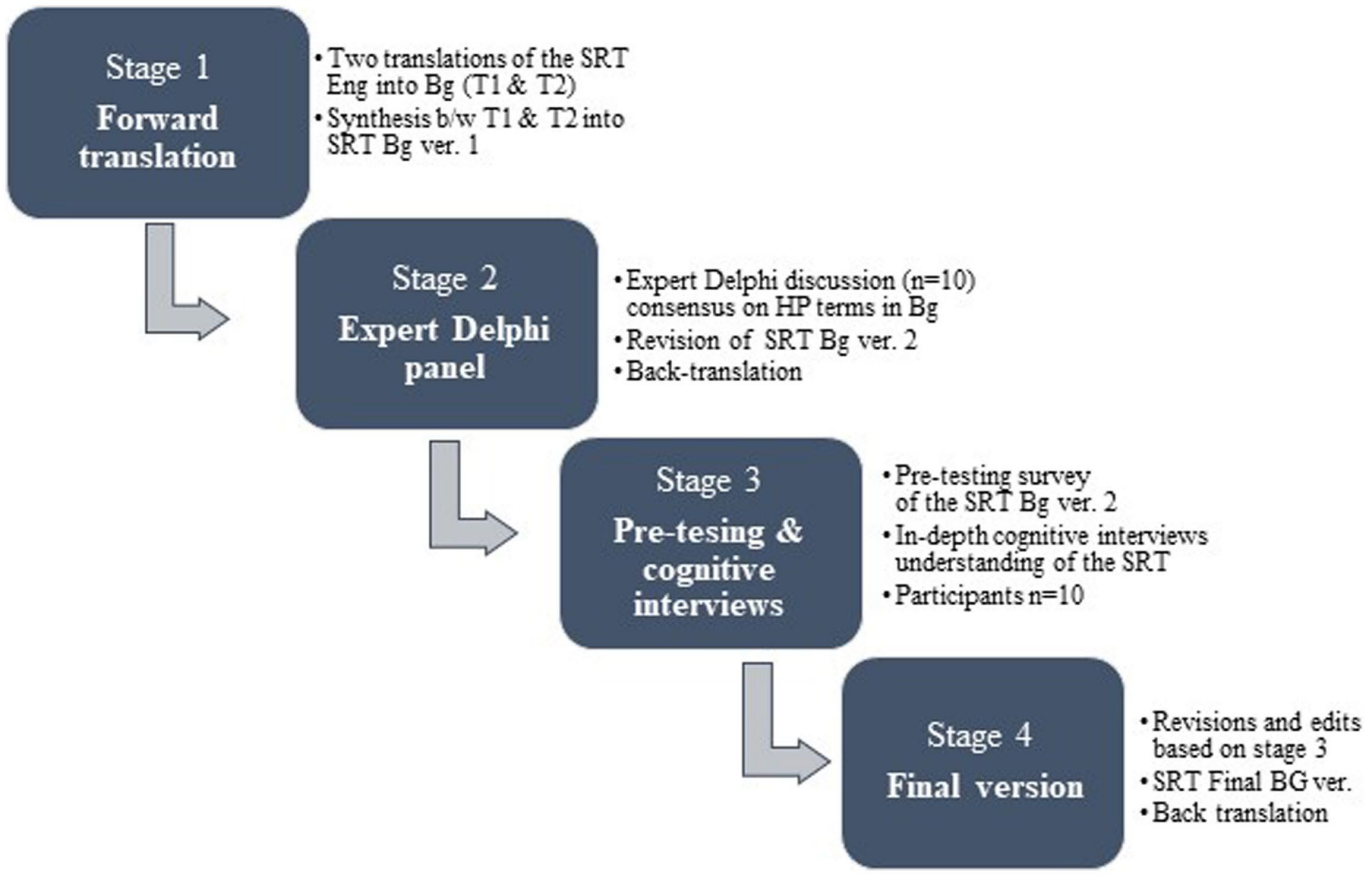
Stages of the cultural adaptation process of the HPU self-review tool in Bulgarian.

The development of the Bulgarian version of the SRT followed the WHO methodology for cultural adaptation of research instruments, aiming at conceptual rather than linguistic equivalence between the original and the final version of the tool in another language (19). This methodology, identified as best practice for cultural adaptation by researchers (20) comprises four successive stages as presented in Figure 1.

#### 2.2.1 Forward translation

Two independent licensed translators, native Bulgarians with excellent knowledge of English language and culture, conducted forward translations of the original instrument from English into Bulgarian. The translations were then independently compared by two public health (PH) researchers and synthesized into one file, the first working Bulgarian version of the SRT.

#### 2.2.2 Expert panel and back-translation

The expert panel was conducted in compliance with the applied WHO methodology, of which it was an integral part. Experts in the fields of PH and HP from one town, but from different health institutions were selected for invitation based on their educational background, research activities, and practical experience. Twelve potential respondents were invited via email to participate in the Delphi expert discussion. Each participant received: (1) a written description outlining the purpose of the Delphi discussion, (2) the original SRT, (3) the two independent Bulgarian translations, (4) the first Bulgarian version of the tool (synthesized from the two translations), and (5) a list of HP expressions, phrases and concepts, for which consensus needed to be reached by the experts regarding their presentation in the Bulgarian version of the tool. The invited specialists had to be fluent in English. The expert panel followed a structured discussion scenario aimed at achieving consensus on the Bulgarian wording of the specific HP terms (21). The outcome of the expert panel’s work was the revised version of the SRT in Bulgarian.

Back translation—The revised instrument was translated back into English. In this case, the translator was not aware of the original instrument and the purpose of the study and was not involved in the first stage of the translations. The translator had to be native English speaker, fluent in Bulgarian. The original and the new English version of the SRT were analyzed and compared for conceptual equivalence.

#### 2.2.3 Pre-testing and cognitive interviewing

The Bulgarian version of the SRT underwent a pre-testing process. Representatives of the target groups (students, academic, administrative and university management staff) were invited and briefed on the purpose, methodology and their role in the study. Informed consent was sought and obtained. This stage occurred in two phases: (1) The SRT was administered to the participants online, through Google forms for independent survey completion (2) in-depth cognitive interviews were conducted with the respondents to gauge their understanding of specific HP terms, phrases, statements through interpersonal communication (22). Interviews were scheduled at the convenience of the participants, typically 7–20 days (with an average of 15 days) after the survey. Participants were asked to assess the clarity of statements, terms or phrases, and provide suggestions for alternative expressions. The duration of interviews ranged from 60 and 120 min, averaging 90 min. Interviews were recorded, transcribed, coded, and analyzed.

#### 2.2.4 Preparation of final instrument version

At this stage, each respondent’s answers to each statement was compared for consistency with survey responses. Each statement was reviewed and compared repeatedly for consistency with the original instrument. Comments, opinions and suggestions from participants in the interviews were taken into account in the final revision of the self-assessment tool in Bulgarian.

Additionally, a reverse translation from Bulgarian to English of the final version of the instrument was conducted. The translated document was provided to the creator of the original tool for review.

The study was conducted after approval from the University Ethics Committee (No 101/ 24.03.2021). Qualitative data analysis was performed using QSR NVivo v. 11 software, and graphical and tabular methods were applied to visualize the results.

## 3 Results

### 3.1 Stage 1 Cultural adaptation during forward translation and synthesis

The cultural adaptation process of the SRT to the Bulgarian context and language started with two independent translations from English into Bulgarian by professional licensed translators both native Bulgarian speakers fluent in English. Conceptual discrepancies arose between the two Bulgarian texts and the original document, which was anticipated given the translators’ lack of familiarity with health promotion terminology. Two researchers independently compared the two Bulgarian translations to identify discrepancies between them and with the original document.

For example, one of the standard answers “Yes, we are there” was translated directly in Bulgarian as “Yes, we are present here,” in one version and as: “Yes, we are active in this direction” in the other. The term “wellbeing” was translated with two different Bulgarian words with distinct meanings. The researchers reached consensus on each problematic expression, selecting the more appropriate translation. This selective synthesis resulted in the initial working Bulgarian Version 1.0 of the instrument, which underwent critical review in a subsequent Delphi expert panel discussion.

### 3.2 Stage 2 Delphi expert panel

The purpose of the Delphi expert panel was to revise the synthesized Bulgarian translation of the SRT to make it suitable for application in a Bulgarian context. The rationale for choosing the method was the necessity for achieving expert consensus on the best Bulgarian conceptual rather than literary translation of specific health promoting terms and concepts used in the original SRT.

Ten out of the twelve invited experts participated in the Delphi panel, with nine women and one man, having a mean age of 52 years (median 14.2). All participants were Bulgarian experts in the field of public health and health promotion with significant research and teaching experience (Table 1). All participants had excellent knowledge of English, regularly teaching disciplines in the field of public health including health promotion in English language. A single round of Delphi discussion was conducted facilitated by the lead researcher. Criteria for consensus were predetermined as 75% agreement on specific wording for each discussed term/concept. The discussion lasted for 90 min.

**Table 1:** Characteristics of the participants in the Delphi expert panel.

Expert	Gender	Age	Research and teaching expertize
R1	Female	70	Health promotion, Public health
R2	Female	56	Health promotion, Public health
R3	Female	51	Public health, Health promotion, Sociology
R4	Female	68	Public health, Biostatistic
R5	Male	40	Health Promotion, Psychology
R6	Female	48	Public health, Health promotion
R7	Female	51	Health promotion, Public health
R8	Female	51	Public health, Health promotion
R9	Female	34	Public health
R10	Female	57	Public health, Health promotion

Ten specific English HP terms were proposed to the experts for discussion. For each term at least one Bulgarian version was proposed (Table 2) with maximum 8 Bulgarian possibilities for the name of the initiative. The name of the initiative “Health promoting universities” was most continuously discussed, followed by the expression “whole system approach,” and the seemingly straightforward English term “wellbeing.” The final consensus on the Bulgarian wording for each term is presented in Table 2.

**Table 2:** Terms discussed and decisions adopted by the expert panel.

Terms in SRT	Number of discussed BG terms	Expert panel consensus in Bulgarian
Health Promoting University/Healthy University	8	Университет за промоция на здравето
Self-review tool	2	Инструмент за самооценка
Wellbeing	3	Благополучие
Welfare	2	Благосъстояние
Whole system approach	3	Цялостен университетски подход
Community	3	Общественост
Local community	2	Местна общност
Mental wellbeing	2	Психично здраве
Engagement	2	Ангажиране
Stakeholders	1	Заинтересовани страни

Following the expert decisions, a second Bulgarian Version of the SRT was developed and translated back into English, by a translator proficient in both English (as a native language), and Bulgarian. The resulting document was assessed by the researchers for conceptual differences with the original instrument. No significant discrepancies were found, leading to the creation of BG Version 2.0 of the SRT, which underwent pilot testing in the subsequent third stage.

### 3.3 Stage 3 pre-test and cognitive interviews

Ten participants were involved in the third stage of the adaptation process—four students, two administrative employees, two representatives of the academic staff and two members of the academic management. The age of the participants ranged from 19 to 53 years (median 34), nine of whom were women.

The participants first independently completed all questions from the BG Version 2.0, followed by cognitive interviews to discuss their difficulties related to the content, questions’ understandability, clarity of proposed answers, and meaning of particular texts.

During the cognitive interviews the interviewer read the questions aloud with appropriate intonation and pauses to aid comprehension. This process revealed some difficulties experienced during the survey phase. A student remarked: “Now that you are reading it to me…” indicating improved understanding. Similarly a teacher mentioned: “…on the second reading…” suggesting enhanced clarity upon hearing the question read aloud.

The interviews not only helped the participants to understand the meaning of specific health promotion concepts, but also prompted them to recall relevant examples that informed their responses. Some remembered existing access for people with disabilities, others—health services provided by the institution related to dental health etc.

Some participants changed their opinion in the period between the self-completion and the interview stages. For instance an employee remarked while thinking aloud: “…but now I answer that…” indicating a shift in perspective. Additionally, after completing the instrument on their own, some participants began noticing existing activities within the university, that they previously overlooked. For example, a lecturer commented: “… then I saw that there are bicycle racks in the courtyards of two university buildings… that’s an encouragement to travel by bike…“highlighting newfound awareness of university initiatives promoting health and wellbeing.

#### 3.3.1 Understandability of HP terms and concepts

During the cognitive interviews specific attention was payed to the understandability of HP terms, both those discussed by the Delphi panel and any additional, identified by the interviewees.

Difficulties persisted with the understandability and clarity of expressions such as “whole system approach.” During the interviews, the Bulgarian translation of “whole system approach” prompted questions about its precise meaning. After clarification by the interviewer, it was understood and accepted by the participants. This indicated that the term was unfamiliar to some respondents. However, once its meaning was explained, none of the respondents suggested a better alternative than the one agreed upon by the Delphi panel.

Similarly the Bulgarian translation of “external stakeholders” raised questions “who exactly are they?.” Examples provided during the conversation helped clarify the term and the respondents suggested similar examples to be included in the text which was considered.

An interviewee from the academic staff highlighted the differentiation between “wellbeing” and “welfare,” emphasizing the economic/financial aspects of welfare. This led to the decision to retain only the Bulgarian term for “wellbeing” in the tool.

Additionally, terms like “sustainable development” required additional clarification. It became evident during the interviews that the content of this term was unclear to respondents not familiar with health promotion and public health. Furthermore, there was a discrepancy in the interpretation of these terms between students and other respondents.

#### 3.3.2 Understandability of other non-specific, but ambiguous terms/words

The term “strategy” whether used alone or in phrases as “strategic links,” or “strategic planning” were perceived as complex. Similarly, the expression “the university ensures” in the context of statements as “the university ensures that health and wellbeing related strategic planning and delivery are inclusive” was initially translated into Bulgarian as similar to “the university guarantees.” However the respondents were unsure how the university could guarantee inclusive strategic health planning and delivery. The term “guarantees” was deemed vague by all participants leading to its replacement with “the university aims to secure…” in Bulgarian.

The statement “free drinking water is available on campus” was understood as implying that bottled mineral water must be provided to the university community at no cost. The expression was described as both misleading and redundant. The statement was revised to “drinking water is accessible in all university buildings” in the final version of the tool.

The statement “the university has standards for accommodation to ensure that health and wellbeing…” led to confusion among participants, particularly regarding the Bulgarian word for “accommodation.” Students interpreted it in the context of the student dormitories, while teachers and administrative staff thought of libraries, teaching rooms, working areas. The wording in the final version was adjusted to focus on the living areas accommodations.

#### 3.3.3 Wording of statements and answers

One consequence of the translation of the original tool was the lengthening of the sentences in the Bulgarian version of the tool. The average number of the words per statement was 22.5 words, and the longest statement comprised 41 words. This led some respondents to perceive the instrument as “complex, written in a high style” and overly lengthy.

Participants agreed that providing examples, or clarifying terms with words in brackets, would enhance understanding. A student commented: “so, giving examples would be better because it makes it clearer to me”; an employee noted: “Yes, here the example suggests….” Some respondents—members of the academic staff and management tried to reason on behalf of another target group: “…I’m not aware of how familiar a student will be”—words of a teacher.

The section “Leadership and governance” was identified as the most difficult for the students, teachers and staff, whereas “Academic, personal, social and professional development” was deemed the easiest, management representatives finding all statements easy to answer.

Issues were reported with the understanding of the standard responses following each statement. The option “Not at all/Do not know” was confusing for many respondents, as it seemed contradictory. A student commented: “It turns out that I disagree…while I actually do not know.” A teacher who disagreed with the wording explained his opinion “It implies simultaneously two very different hypothesis: either the university is not doing anything about an issue, or it is active but the respondent is not aware about it.” Separating the two parts of this answer into distinct options addressed this confusion. Some respondents found it difficult to distinguish between the responses “Working on this currently”; and “Thinking about it.” Some of the comments implied that the thinking process in the institution was a step from the working process. So, the final version of the Bulgarian instrument had the following standard answers: Yes (corresponding to “Yes we are there”); To some extent (corresponding to “Working on this currently”); No; Do not know.

#### 3.3.4 Limited applicability of certain statements

Certain statements in the SRT were deemed not directly applicable to certain respondents. For example, students found it difficult to express opinions about opportunities for professional development offered to teachers and staff. Students, faculty and administrative staff had lower awareness about university policy, strategic planning and budgeting issues than academic leadership respondents.

In total 61 (89.7%) of 68 statements in the working BG Version 2.0 were revised, based on the results of the testing. The changes mainly focused on simplifying wording, reordering words, replacing ambiguous terms, and eliminating misleading or redundant phrases. The final version, designated as BG Version 3.0, was then used for the self-assessment process.

## 4 Discussion

Тhis paper describes the systematic cultural adaptation process of a specific instrument designed for self—assessment of a health-promoting higher educational institution (university, campus, college etc.) referred to as the SRT. Our aim was to develop a conceptually relevant Bulgarian version of the instrument suitable for a culture different from the original western European context in which the tool was initially developed and applied (13). While the SRT had been applied in institutions outside the UK, it had not been culturally adapted for non-English speaking cultures until this study.

The cultural adaptation of the HPU Self-Assessment Tool in Bulgarian was justified and initiated based on previous research indicating the applicability and effectiveness of international documents in the field of HP, including in Bulgaria (18, 23).

It was recognized that such cultural adaptation is not only important, but also essential, as even translations edited by experts, as in the expert Delphi panel in this study, could not ensure full understanding of HP-related texts by the wider university community.

Despite following an internationally recognized methodology for cultural adaptation, the process did not progress quickly and smoothly. This could be primarily attributed to the lack of familiarity with HP concepts in Bulgaria such as the “healthy settings initiative” and with the role of educational environments in promoting health, particularly the HPU. Additionally, there was a scarcity of strategic HP documents in Bulgarian and recognizable real-life examples, as, e.g., the HP schools, which could have served as a foundation for the HPU initiative (24).

According to leading HP experts in our country, the adoption of the philosophy of HP in Bulgaria is accompanied by a number of difficulties. One of them is related to specificities of the political culture across all social domains, characterized by a high degree of centralization of management and high-power distance between top and lower levels in institutions, including educational ones (18). On the other hand, there are barriers related to the interpretation of the concept of HP itself.

Created in countries with Western European culture, the terminology is difficult to adapt to the Bulgarian language and culture (18). Even today the HP concepts are defined as insufficiently theoretically developed and practically applied in Bulgaria (25).

Pinto et al. (26) note that HP instruments developed on the basis of generally accepted HP principles need to be adapted to the specific cultural characteristics before their application in diverse national contexts.

In our case the entire cultural adaptation process of the SRT took approximately 10 months. A similar duration was reported for the adaptation of a smoking prevention questionnaire to the Bulgarian language (18) which is comparable to reports by other authors (27).

Some studies validating foreign instruments, report no major discrepancies between the first-stage translations and the original document (28). Others like ours find poor comparability between the translations and the original texts, attributing this to the translators’ focus on wording rather than meaning (29).

All stages of the methodology applied by us proved to be important for achieving the intercultural adaptation. The work of the Delphi Expert panel and the pilot-test stage with the participation of representatives from the entire university community were particularly useful. Our experience is supported by other researchers. Oliveira and Bandeira (30) identified these two phases as significant for the successful adaptation of a psychological instrument assessing personality structure and organization to Brazilian culture and traditions.

Expert input was essential for the adaptation of HP terminology but insufficient. It was found that even after the decisions of the Delphi panel, difficulties in understanding some terms and concepts, especially among students persisted. This might be attributed, on one hand, to the professional bilingual competence of the experts. Such hypothesis was proposed by Norwegian researchers working on the cultural adaptation of an English language instrument (29). Experts fluent in English more easily understand the concepts in their language, which does not automatically apply to a monolingual target population (29). On the other hand, while experts use HP terms freely and routinely, these terms are rarely present in daily Bulgarian speech, explaining the differential understanding between experts and the wider audience.

Thus, testing among representatives of the university community proved to be a particularly useful procedure. The active participation, comments and suggestions of the participants greatly aided the cultural adaptation in our research. This was also confirmed by Oliveira and Bandeira (30), according to whom this stage led to the clarification of confusing and misinterpreted elements. Squires et al. (31) described the cultural adaptation of an instrument in 12 European countries, in 11 languages. For the purpose of their study, the adaptation methodology included translations of the original instrument into the target languages and expert panels were held three times. It was found that in one of the countries, Poland, the process of cultural adaptation was impossible without pilot testing, which turned out to be the key success factor.

The apparent difficulties with testing the instrument among some respondents led to the conclusion that not all statements are equally applicable to all participants. According to Chengelova (32), survey questions should correspond to the competence and level of awareness of the social group to which respondents belong. The creators of the original SRT recommended that it should be completed not individually but by a team of representatives from the entire university community (11). Thus, various statements would receive answers from respondents who are competent to provide an opinion on behalf of their target group.

The cultural adaptation of the SRT in our study resulted in a change in 61 of the total 68 statements. Similar results were reported by Lopatina et al. (33), discussing the adaptation of a WHO health literacy assessment tool for Russian-speaking populations in Germany, Israel, Russia, Kazakhstan, and the United States. The linguistically and culturally adapted instrument in Russian was obtained after changing 95% of the questions.

An interesting, but also sought after and expected effect of our research was the popularization of the HPU initiative among the study participants. They shared their positive attitude because their opinion was sought and valued. Respondents showed interest and paid attention to health promotion activities at the university that they had not noticed before.

One possible limitation of our study is the convenience sampling of respondents in the Delphi expert panel. However, the choice of participants could not affect the content of the questionnaire, as the discussion focused on achieving the best possible Bulgarian wording for all the questions from the international instrument, rather than determining which questions to include. A second limitation is that the study was conducted within a specific higher education institution predominantly offering teaching in health and medical specialties and disciplines. However, this does not limit the opportunities for the proposed instrument to be tested in other organizations. On the positive side, we can highlight that this is the first systematic cultural adaptation of a self-assessment tool for the HPU in Bulgarian language.

## 5 Conclusion

The presented cultural adaptation process of the HPU self-assessment tool confirms earlier experience that the cross-cultural adaptation of theoretical HP documents to the Bulgarian language and culture is a difficult but an essential process. It creates prerequisites for the ideas, concepts and practical experiences of established HPU to be adopted and developed in Bulgarian higher education institutions. Such a process should always be considered in situations aimed at the application of public health or health promotion policies, strategic documents or instruments in societies with cultures different from the Western Europe and languages other than English.

What follows from now on? We hope that the “HPU Self-Assessment Tool” in Bulgarian will be implemented in other Bulgarian universities to assess their potential to become part of the HPU initiative.

## Data availability statement

The original contributions presented in the study are included in the article/supplementary material, further inquiries can be directed to the corresponding author.

## Ethics statement

The studies involving humans were approved by Комисия по етика на научните изследвания (КЕНИ) на МУ Варна/Committee on ethics of scientific research of the Medical University of Varna. The studies were conducted in accordance with the local legislation and institutional requirements. The participants provided their written informed consent to participate in this study.

## Author contributions

PB: Conceptualization, Data curation, Formal analysis, Investigation, Methodology, Project administration, Writing – original draft, Writing – review & editing. KD: Conceptualization, Formal analysis, Methodology, Supervision, Writing – review & editing.
